# The Small Molecule H89 Inhibits *Chlamydia* Inclusion Growth and Production of Infectious Progeny

**DOI:** 10.1128/IAI.00729-20

**Published:** 2021-06-16

**Authors:** Karissa J. Muñoz, Kevin Wang, Lauren M. Sheehan, Ming Tan, Christine Sütterlin

**Affiliations:** a Department of Developmental and Cell Biology, University of California, Irvine, California, USA; b Department of Microbiology and Molecular Genetics, University of California, Irvine, California, USA; c Department of Medicine, University of California, Irvine, California, USA; Yale University School of Medicine

**Keywords:** intracellular infection, developmental cycle, RB-to-EB conversion, isoquinoline sulfonamide

## Abstract

*Chlamydia* is an obligate intracellular bacterium and the most common reportable cause of human infection in the United States. This pathogen proliferates inside a eukaryotic host cell, where it resides within a membrane-bound compartment called the chlamydial inclusion. It has an unusual developmental cycle, marked by conversion between a replicating form, the reticulate body (RB), and an infectious form, the elementary body (EB). We found that the small molecule H89 slowed inclusion growth and decreased overall RB replication by 2-fold but caused a 25-fold reduction in infectious EBs. This disproportionate effect on EB production was mainly due to a defect in RB-to-EB conversion and not to the induction of chlamydial persistence, which is an altered growth state. Although H89 is a known inhibitor of specific protein kinases and vesicular transport to and from the Golgi apparatus, it did not cause these anti-chlamydial effects by blocking protein kinase A or C or by inhibiting protein or lipid transport. Thus, H89 is a novel anti-chlamydial compound that has a unique combination of effects on an intracellular *Chlamydia* infection.

## INTRODUCTION

*Chlamydia* is a genus of pathogenic bacteria of high importance to public health. In the United States, more than 1.8 million cases of chlamydial infection are reported to the CDC each year (1). Almost all of these cases are genital infections due to Chlamydia trachomatis, which is the most common etiology of bacterial sexually transmitted disease (2). C. trachomatis also causes trachoma, which is the most common form of infectious blindness in the world (2). A related species, C. pneumoniae, causes community-acquired pneumonia.

All *Chlamydia* species are obligate intracellular bacteria that share an unusual biphasic developmental cycle (3). The elementary body (EB) is the infectious form that binds and enters a eukaryotic host cell, where it remains within a membrane-bound vacuole called the chlamydial inclusion. By 2 to 8 h postinfection (hpi), the EB converts into a reticulate body (RB), the replicating, but noninfectious, form of the bacterium. Expansion of the RB population through multiple rounds of division is followed by asynchronous conversion of individual RBs into EBs. This conversion begins at about 24 hpi for C. trachomatis with the appearance of conversion intermediates called intermediate bodies (IBs), which then become EBs (4). IBs and EBs are morphologically different from RBs, and these three developmental forms can be distinguished by electron microscopy (EM). Conversion is marked by expression of late chlamydial genes that encode EB-specific proteins. Between 40 and 72 hpi, depending on the species, EBs are released from the host cell by sequential lysis of the inclusion and host cell (5) or by extrusion of the inclusion from an intact host cell (6).

The chlamydial inclusion is a dynamic organelle whose membrane is made up of host-derived lipids and chlamydial proteins. It originates from an endocytic vesicle that expands 1,000-fold in volume during the intracellular infection (4). This dramatic growth in inclusion volume and membrane surface area depends on the acquisition of lipids from the host cell (7–9). The inclusion membrane also contains about 50 chlamydial proteins, called Incs, which are integral membrane proteins that mediate interactions with the host cell (10, 11). Host proteins have been detected in the vicinity of the inclusion (7, 12, 13), but only a few reports document their insertion into the inclusion membrane (14).

*Chlamydia* obtains lipids by hijacking membrane-trafficking pathways of the host cell (7, 15–17). For example, in an infected cell, post-Golgi vesicles are rerouted to deliver host sphingolipids and cholesterol, but not proteins, to the inclusion and the bacteria themselves (18–20). Vesicles that mediate anterograde and retrograde transport between the endoplasmic reticulum (ER) and the Golgi apparatus are also important for the infection (21–23). However, it is unclear how these vesicular trafficking pathways are diverted during the intracellular *Chlamydia* infection and how they deliver host lipids to the inclusion.

The small molecule H89 has been used as a tool for investigating vesicular transport pathways. This isoquinoline sulfonamide was initially identified as a selective inhibitor of protein kinase A (PKA), although it has also been found to inhibit a number of other cellular serine/threonine kinases (24–29). H89-mediated disruption of PKA activity blocks post-Golgi apparatus transport, but the negative effects of this compound on ER-to-Golgi apparatus transport involve an unidentified host kinase (30–32).

In this study, we used H89 as a pharmacological tool to better understand the host cell pathways that contribute to inclusion growth and progeny production during a *Chlamydia* infection. We show that H89 treatment of C. trachomatis-infected cells slowed inclusion growth and altered the chlamydial developmental cycle by reducing both RB replication and RB-to-EB conversion. Mechanistic analyses revealed that these phenotypes were not caused by inhibiting PKA or protein kinase C (PKC) activity or by disrupting vesicular transport of host lipids or proteins.

## RESULTS

### H89 slows the growth of the chlamydial inclusion.

**FIG 1:**
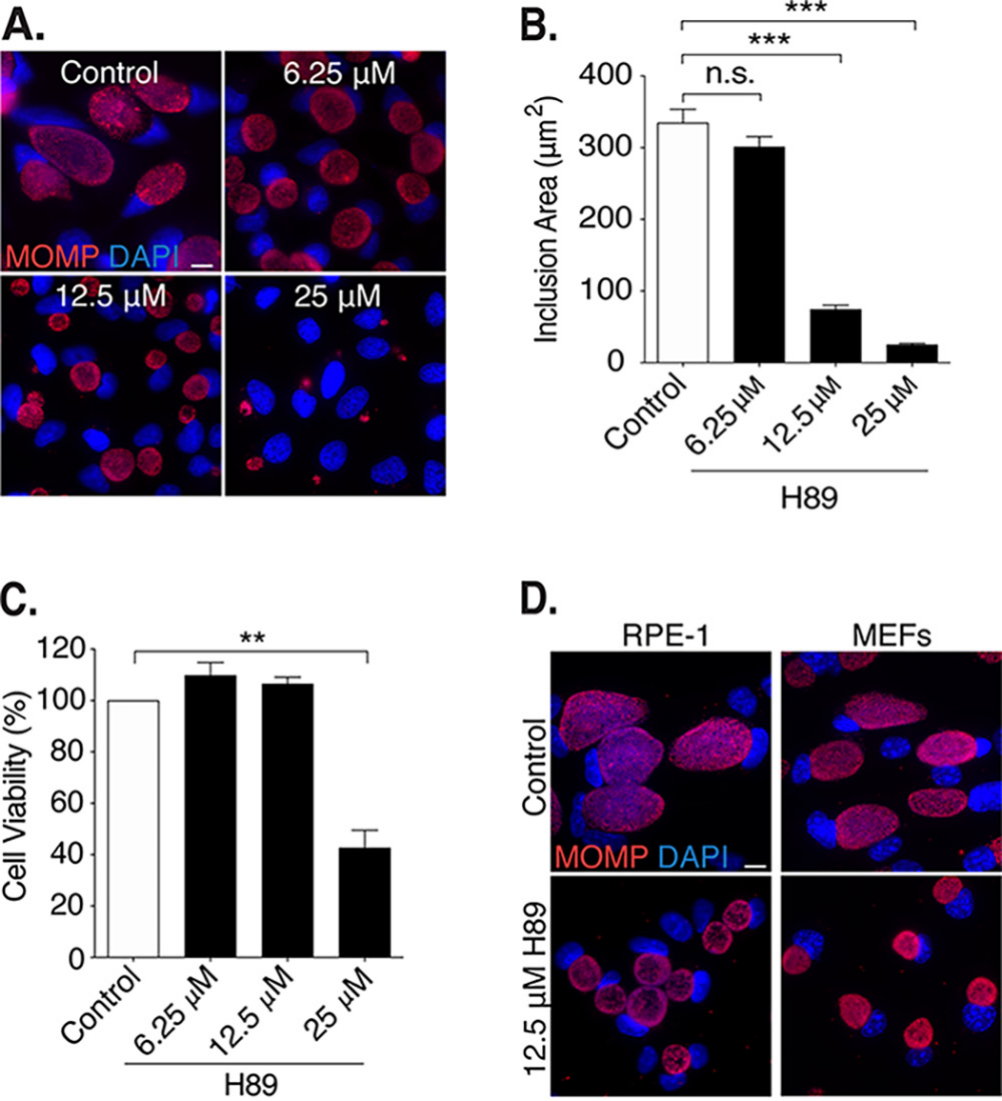
H89 decreases C. trachomatis inclusion size. (A) HeLa cells grown on coverslips were infected with C. trachomatis L2 at an MOI of 3 and treated from 1 to 32 h postinfection (hpi) with the indicated concentrations of H89. Control cells (control) were incubated in the equivalent volume of DMSO, which served as the solvent for H89. In these immunofluorescence images, chlamydiae are detected with antibodies to the major out membrane protein MOMP (red) and chlamydial and host DNA are stained with DAPI (blue). Scale bar, 10 μm. (B) Quantification of the inclusion areas of the cells in panel A. One hundred inclusions were measured for each condition. Data from one representative experiment (*n* = 3) are shown as means ± SD; ***, *P < *0.001. n.s. indicates the data are not statistically significant. (C) Viability of uninfected HeLa cells incubated with different concentrations of H89 for 32 h was assessed with an MTT cell viability assay. Data were normalized to the viability of untreated cells and are expressed as a percentage. Data are presented as means ± SD (*n* = 3); **, *P ≤ *0.01. (D) Immunofluorescence images of retinal pigment epithelial cells (RPE-1) and mouse embryonic fibroblasts (MEFs) grown on coverslips, infected with C. trachomatis L2 at an MOI of 3, and treated with 12.5 μM H89 from 1 to 32 hpi. Scale bar, 10 μm.

We found that H89 treatment of *Chlamydia*-infected cells produced a concentration-dependent reduction in inclusion size (Fig. 1A and B). At 32 hpi, 25 μM H89 decreased inclusion area by 92% compared to control cells treated with vehicle, but this high concentration of H89 had significant cytotoxic effects on the host cell (Fig. 1C). H89 at 6.25 μM, in contrast, had no significant effect on inclusion size (Fig. 1A and B). Instead, we used 12.5 μM H89 for all experiments described in this study, because it reduced inclusion size by 78% without affecting cell viability or the percentage of host cells with an inclusion, which is a measure of infection efficiency (Fig. 1A to C; see also Fig. S1A in the supplemental material). H89 at 12.5 μM also decreased C. trachomatis inclusion size in retinal pigment epithelial (RPE-1) cells and in mouse embryonic fibroblasts (MEFs), demonstrating that this phenotype is not cell type specific (Fig. 1D).

**FIG 2:**
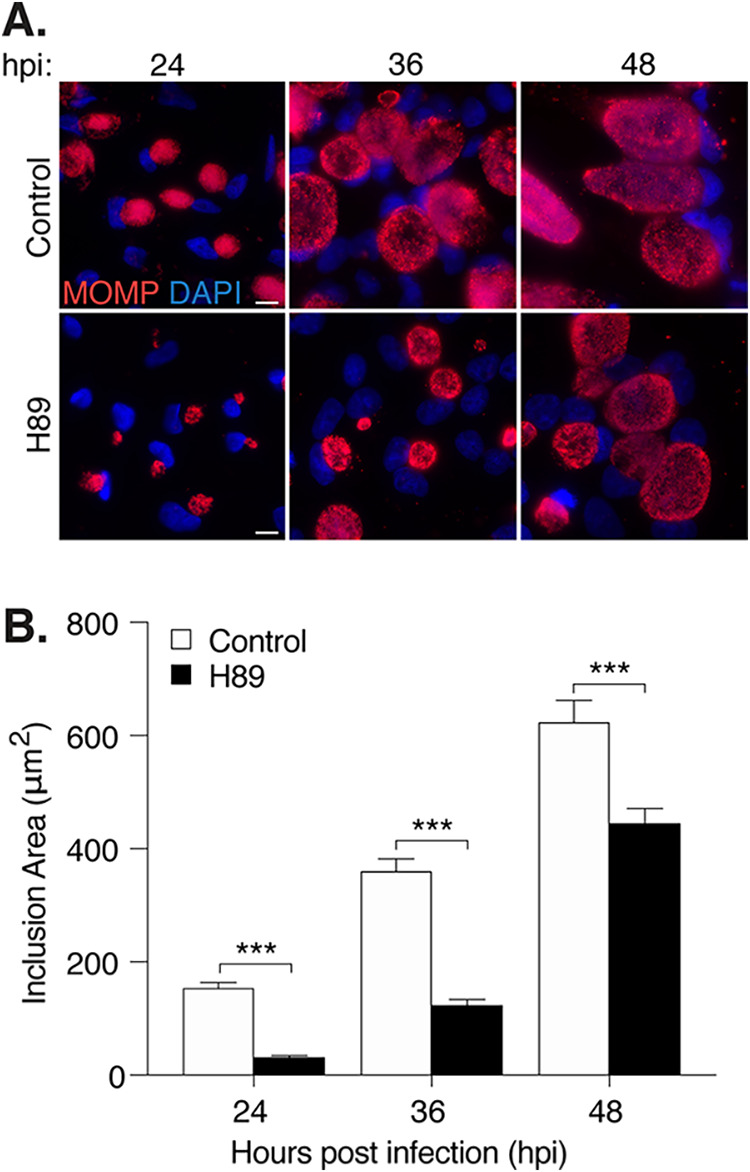
H89 delays *Chlamydia* inclusion growth. (A) Immunofluorescence images of Chlamydia trachomatis L2-infected HeLa cells treated with H89 starting at 1 hpi. Samples were fixed at the indicated time points. Chlamydiae were stained with antibodies to MOMP (red), while chlamydial and host DNA was detected with DAPI (blue). Scale bar is 10 μm. (B) Quantification of inclusion surface areas for the cells in panel A. One hundred inclusions were measured for each condition. Data from one representative experiment are presented as means ± SD (*n* = 3); ***, *P < *0.001.

We next investigated how H89 alters inclusion growth during the course of the *Chlamydia* infection. We measured the greatest effect at 24 hpi, when inclusions were 5-fold smaller than that in untreated control cells (Fig. 2A and B). H89 also produced significantly smaller inclusions at 36 and 48 hpi. However, inclusions of H89-treated cells continued to grow so that at 48 hpi the difference in size was only 1.4-fold (Fig. 2A and B) compared to that of the control cells. To ensure that H89 was effective throughout the treatment period, we replenished the compound at 24 hpi but observed a similar reduction in inclusion size, as in nonreplenished samples (Fig. S1B). These results indicate that H89 slows, but does not completely block, inclusion growth, and that H89 is stable for the duration of the intracellular *Chlamydia* infection.

### H89 alters the chlamydial developmental cycle.

**FIG 3:**
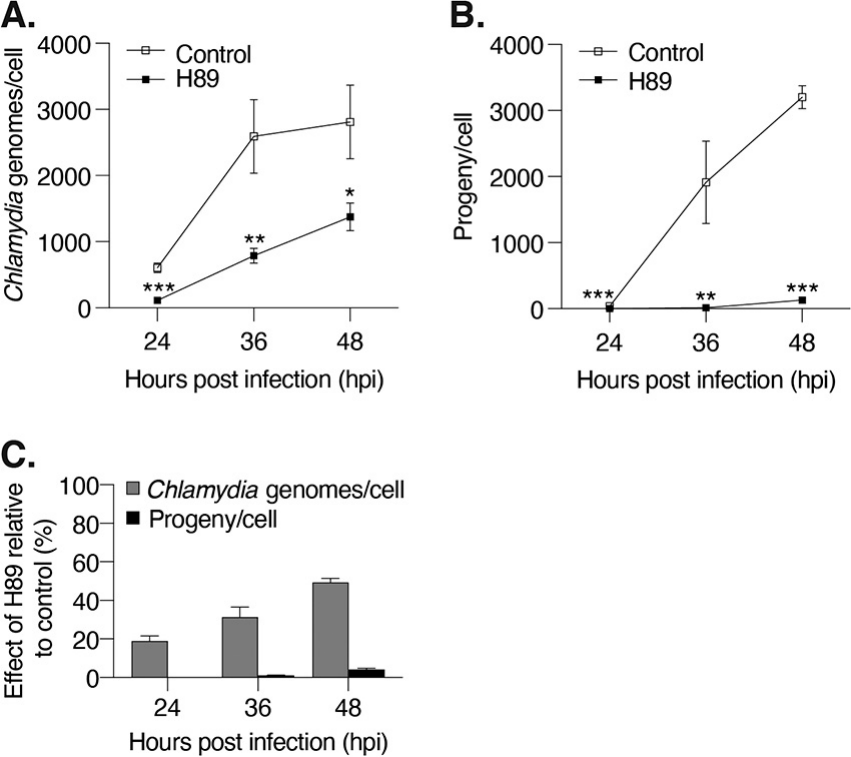
H89 causes a greater effect on infectious progeny than on chlamydial replication. (A) The number of chlamydial genomes in infected, H89-treated HeLa cells was determined via qPCR at the indicated time points and normalized to the number of host cells. Data are presented as means ± SD (*n* = 3). **, *P ≤ *0.01; ***, *P ≤ *0.001. (B) The number of infectious EBs was determined in a progeny assay for infected control and H89-treated HeLa cells at the indicated time points and normalized to the number of host cells. Data are presented as means ± SD (*n* = 3); **, *P ≤ *0.01; ***, *P < *0.001. (C) The numbers of chlamydial genomes and infectious progeny in H89-treated samples were normalized to their untreated control counterparts (control = 100%) and expressed as a percentage to indicate relative effect of H89.

We performed a series of experiments to examine the effect of H89 on *Chlamydia* development. We first used quantitative PCR (qPCR) to quantify the number of chlamydial genomes within each infected cell as a measure of chlamydial replication. Compared to control cells, H89 treatment decreased the number of chlamydial genomes from 12 to 48 hpi (Fig. 3A and Fig. S2A). At the late time of 48 hpi, the number of chlamydial genomes in H89-treated cells was 2-fold lower than that in control cells, indicating that this inhibitor reduced chlamydial replication by a small but measurable amount.

As qPCR does not distinguish between RBs and EBs, we used progeny assays to measure the effect of H89 on the production of infectious EBs. Prior to 24 hpi, the number of progeny with or without H89 treatment was very small (<1 inclusion-forming units [IFU]/cell; data not shown). However, starting at 24 hpi, which is the time when EBs are first detected (4), H89 greatly decreased the production of progeny. For example, at 36 hpi, H89 treatment caused a 112-fold reduction from 1,914 to 17 progeny/cell, and at 48 hpi, H89 treatment caused a 25-fold reduction from 3,200 to 130 progeny/cell (Fig. 3B). Replenishing H89 at 24 hpi did not have additional inhibitory effects on progeny (Fig. S3), further confirming that H89 retained its activity over the course of the infection. Thus, our results show that the effects of H89 on infectious progeny are more than 10-fold greater than those on total chlamydial numbers (Fig. 3C).

**FIG 4:**
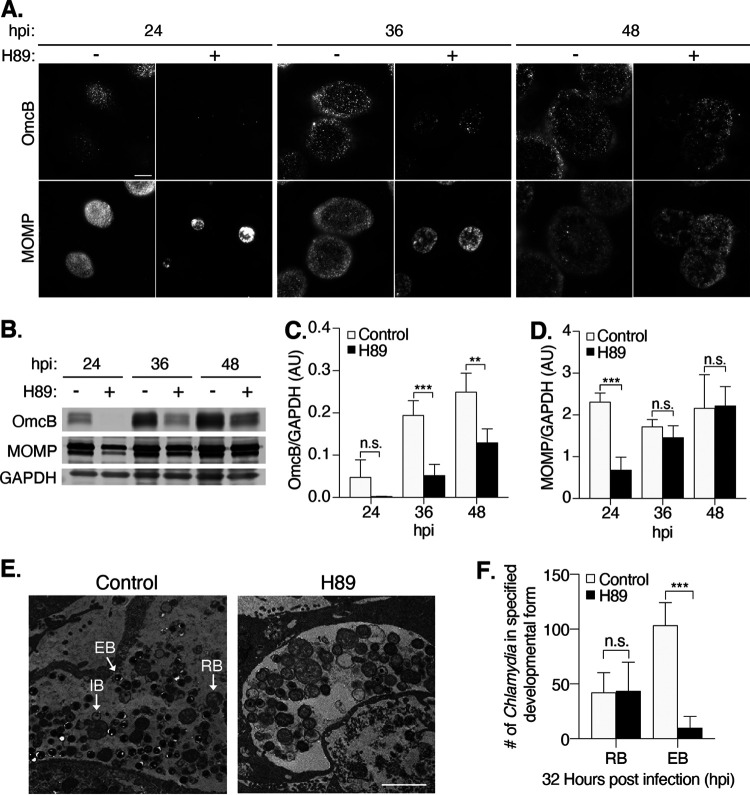
H89 delays expression of an EB-specific protein and reduces the number of EBs. (A) C. trachomatis L2-infected HeLa cells were treated with H89 at 1 hpi and imaged at the indicated time points. EBs were stained with antibodies to OmcB, while all chlamydial developmental forms were detected with antibodies to MOMP. Scale bar, 10 μm. (B) Western blot analysis of total cell lysates from infected cells treated as described for panel A. The levels of OmcB and MOMP are shown. GAPDH served as a loading control. A representative blot is shown (*n* = 4). (C) Quantification of OmcB protein levels from the Western blots in panel B. OmcB levels were normalized to the GAPDH loading control. The data are presented as arbitrary units (AU) and means ± SD (*n* = 4); **, *P ≤ *0.01; ***, *P ≤ *0.001. n.s. indicates that the data are not statistically significant. (D) Quantification of MOMP protein levels from the Western blots in panel B. MOMP levels were normalized to the GAPDH loading control. Data are presented as arbitrary units (AU; mean ± SD) (*n* = 4); ***, *P ≤ *0.001. n.s. indicates the data are not statistically significant. (E) Electron micrographs (EM) of infected cells untreated or treated with H89 from 1 to 32 hpi. Scale bar, 2 μm. The different chlamydial developmental forms are indicated: EB, elementary body; IB, intermediate body; and RB, reticulate body. (F) The numbers of EBs and RBs, detected by EM in 32-hpi inclusions of control and H89-treated infections, were quantified. The data are presented as means ± SD for control (*n* = 7 inclusions) and H89 (*n* = 8 inclusions) treatments; ***, *P ≤ *0.001; n.s., not statistically significant.

We next investigated if H89 causes this disproportionate reduction in infectious chlamydiae by disrupting RB-to-EB conversion. As chlamydial late genes are expressed during RB-to-EB conversion (33), we monitored the expression of the late gene product OmcB, which is an outer membrane protein only present in EBs (34, 35). Immunofluorescence microscopy analysis detected fewer OmcB-positive foci in inclusions of H89-treated cells than in controls at all three time points (Fig. 4A). Similarly, Western blots showed that OmcB levels, after normalization to the loading control, glyceraldehyde-3-phosphate dehydrogenase (GAPDH), were reduced in H89-treated cells by 73% at 36 hpi and 48% at 48 hpi (Fig. 4B and C). Protein levels of MOMP, an outer membrane protein expressed by all chlamydial forms, were not significantly reduced by H89 at these time points when normalized to GAPDH (Fig. 4B and D). H89 also reduced the expression of two other late gene products, Hc1 and Hc2, which are both involved in DNA condensation and EB production (Fig. S4) (34). These results provide evidence that H89 decreases the expression of late chlamydial genes.

As a complementary method to assess RB-to-EB conversion and EB production, we examined the effect of H89 on C. trachomatis-infected cells by electron microscopy (Fig. 4E). At 32 hpi, control cells contained a mixture of RBs, IBs, and EBs, as previously noted (4). In contrast, H89-treated cells and control cells had equivalent numbers of RBs but a 10-fold reduction in EBs (Fig. 4F). We did not detect aberrant bodies, which are abnormally large RBs present during the altered growth state of chlamydial persistence, which can be induced by tryptophan deprivation or antibiotic exposure (36). We conclude that H89 reduces the number of infectious progeny by limiting RB-to-EB conversion.

### H89 treatment at 12.5 μM does not alter vesicular trafficking of proteins or lipids in *Chlamydia*-infected cells.

We next examined if the anti-chlamydial effects of H89 were due to disruption of ER-to-Golgi and post-Golgi apparatus transport. For these studies, we used C1 HeLa cells, in which protein trafficking can be easily observed because they stably express the green fluorescent protein (GFP)-tagged FM4-hGH transport reporter (37). FM4-hGH accumulates in the ER, but the addition of the D/D solubilizer promotes transport of the reporter from the ER to the Golgi apparatus and then to the plasma membrane for secretion.

**FIG 5:**
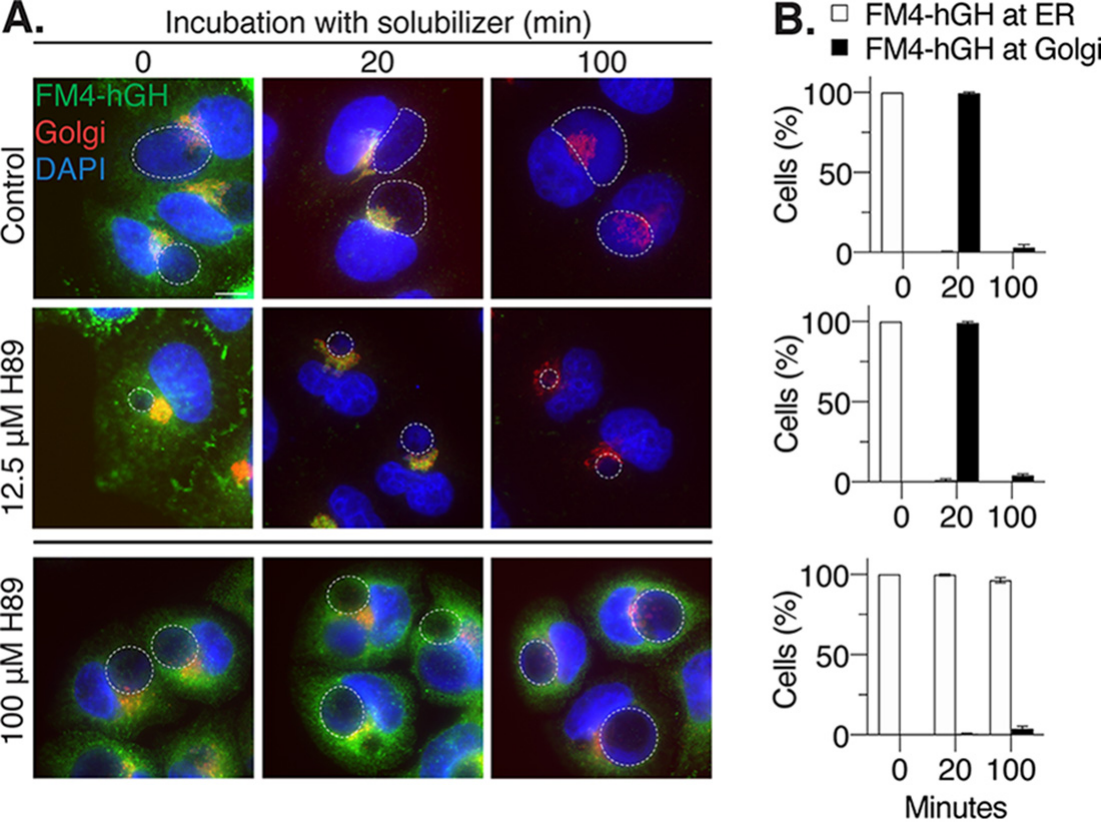
H89 at 12.5 μM does not alter ER-to-Golgi or post-Golgi apparatus trafficking in *Chlamydia*-infected cells. (A) C1 HeLa cells, which express the GFP-tagged transport reporter protein FM4-hGH (green), were infected and treated with H89 at 1 hpi. At 32 hpi, cells were incubated with solubilizer for 0, 20, or 100 min and then fixed and processed for fluorescence microscopy analysis. The Golgi apparatus was visualized with antibodies to GM130 (red), and chlamydial and host DNA were stained with DAPI (blue). The bottom panel shows a control sample in which infected C1 HeLa cells were treated with 100 μM H89 from 30 to 32 hpi before the addition of solubilizer at 32 hpi. Chlamydial inclusions are outlined with white, dashed lines. Scale bar, 10 μm. (B) The number of cells with GFP-tagged FM4-hGH at either the ER or the Golgi apparatus was normalized to the control and expressed as a percentage for the indicated time points after solubilizer addition (minutes). The data are presented as means ± SD (*n* = 3).

H89 at 12.5 μM did not alter protein trafficking in *Chlamydia*-infected C1 HeLa cells, as determined by fluorescence microscopy. At time zero, each condition had 100% GFP reporter localization at the ER (Fig. 5A and B). In nearly 100% of control and H89-treated cells, the GFP reporter left the ER after solubilizer addition and reached the Golgi apparatus by 20 min (Fig. 5A and B). After 100 min, the reporter was released from the cell, as measured by loss of the GFP signal (Fig. 5A and B). In contrast, FM4-hGH remained in the ER in >97% of cells treated with 100 μM H89, a concentration that is known to block protein trafficking (30) (Fig. 5B). To control for the smaller inclusion size of H89-treated cells, we also compared H89-treated cells at 36 hpi with untreated control cells at 24 hpi (Fig. S5A and B) and found that protein secretion occurred independently of the size of inclusions in H89-treated cells.

We also tested if 12.5 μM H89 disrupts the vesicular delivery of lipids to the inclusion and chlamydiae. In a well-established ceramide transport assay, the fluorescent ceramide analog C_6_-NBD ceramide is converted into C_6_-NBD sphingomyelin in the Golgi apparatus and then incorporated into the inclusion membrane and chlamydiae (19, 20). To account for the reduced inclusion size of H89-treated cells, we performed this assay at 36 hpi for H89-treated samples and 24 hpi for controls (Fig. S6). At 0 min, C_6_-NBD ceramide was in the cytosol, only faintly present in the Golgi apparatus, and absent from the inclusion. After 20 min, fluorescent sphingomyelin was at the Golgi apparatus but was also detected in bacterial membranes of control and H89-treated cells. As a positive control, we showed that brefeldin A (19), which inhibits trafficking to the inclusion, completely blocked C_6_-NBD ceramide delivery. We conclude that 12.5 μM H89 is unlikely to block inclusion growth and EB production by disrupting host protein or lipid transport pathways.

### H89 does not inhibit PKA or PKC activity in *Chlamydia*-infected cells.

**FIG 6:**
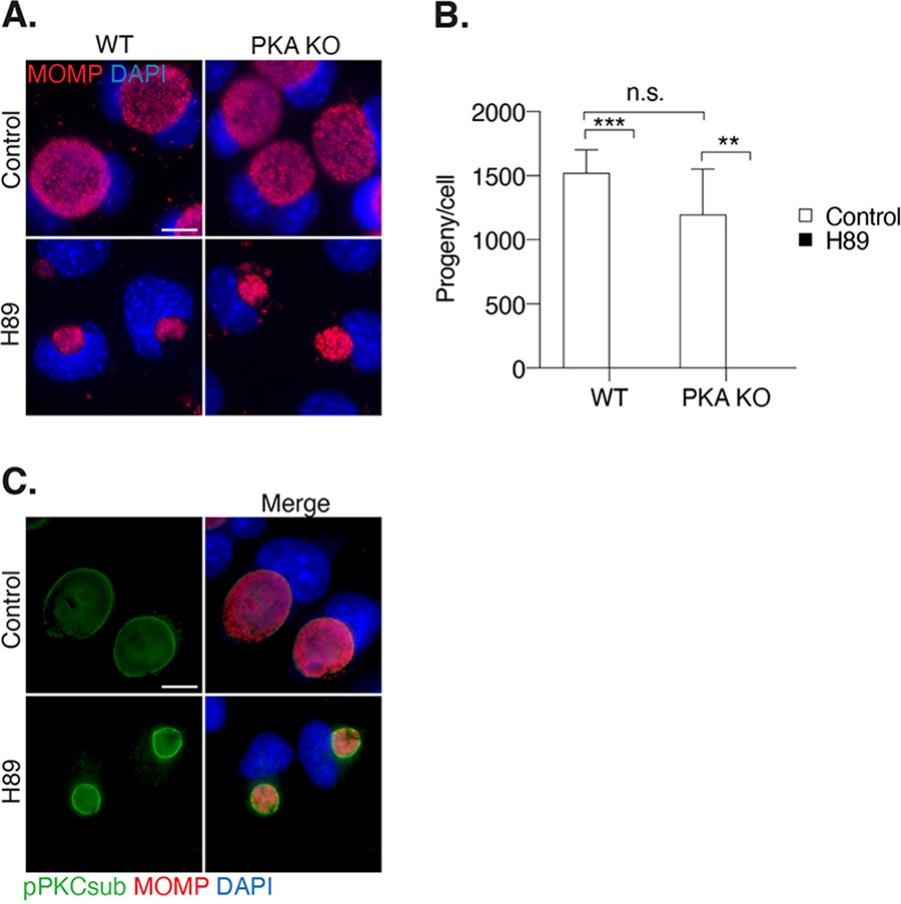
H89 effects on *Chlamydia* development are not due to inhibition of PKA or PKC. (A) mpkCCD WT and PKA knockout (PKA KO) cell lines were infected with *C. trachomatis* L2 and either left untreated or treated with 12.5 μM H89 from 1 to 32 hpi. Cells were fixed and subjected to immunofluorescence microscopy analysis with antibodies to MOMP (red). Chlamydial and host DNA were stained with DAPI (blue). Scale bar, 10 μm. (B) Production of infectious EBs (progeny assay) was measured for the cells treated as described for panel A, normalized to the number of host cells, and presented as means ± SD (*n* = 3); **, *P ≤ *0.01; ***, *P < *0.001; n.s., not statistically significant. (C) Immunofluorescence images of *C. trachomatis* L2-infected HeLa cells fixed at 24 hpi and stained with an antibody that recognizes phosphorylated PKC substrates (green). Chlamydiae were detected with antibodies to MOMP (red). Chlamydial and host DNA were stained with DAPI (blue). Scale bar, 10 μm.

We tested if H89 altered chlamydial infection through its known inhibitory effects on protein kinase A (PKA). For this experiment, we compared *C. trachomatis* infection in the stable PKA knockout cell line mpkCCD (KO) and its wild-type (WT) counterpart (27). H89 caused a similar decrease in inclusion size (Fig. 6A) and progeny (Fig. 6B) for both PKA KO and wild-type cells. We verified that infection efficiency in PKA KO cells was similar to that in wild-type cells (data not shown).

We also investigated if H89 has negative effects on *Chlamydia* infection by inhibiting protein kinase C (PKC), another known H89 target in host cells. PKC is relevant to the *Chlamydia* infection because phosphorylated PKC substrates have been detected at the inclusion membrane (25, 38). However, immunofluorescence microscopy analysis showed that phosphorylated PKC substrates were still present at the inclusion in H89-treated cells (Fig. 6C). Thus, the H89-induced phenotypes in *Chlamydia*-infected cells are unlikely to be caused by inhibition of PKA or PKC activity.

## DISCUSSION

In this study, we examined the effects of the small molecule H89 on intracellular *Chlamydia* infection. We found that this compound slowed inclusion growth and disrupted the chlamydial developmental cycle. H89 decreased the production of infectious progeny by causing a small reduction in RB replication and a much larger decrease in RB-to-EB conversion. The effects of H89 on the infection were not caused by inhibiting two known kinase targets or vesicular transport pathways.

The initial goal of this study was to use a pharmacological approach to examine the role of host vesicular transport during chlamydial infection. We chose H89, because this small molecule disrupts vesicular transport to and from the Golgi apparatus in uninfected cells at concentrations of up to 100 μM and incubation times of up to 2 h (30–32). However, H89 treatment at these high concentrations over the 2-day course of the intracellular C. trachomatis infection was toxic to the host cell. Therefore, we used a concentration of 12.5 μM, which had no cytotoxic effects on the host cell and did not interfere with protein and lipid transport, but still altered both the chlamydial inclusion and developmental cycle.

Our data indicate that H89 slows, but does not abolish, inclusion growth. The treatment with this compound decreased inclusion size at all time points measured. The greatest size difference was at 24 hpi, but the inclusion size gradually caught up so that there was only a modest 1.4-fold difference at 48 hpi. A defect in host lipid acquisition is known to interfere with inclusion growth. While we showed that H89 did not alter sphingomyelin transport to the inclusion, we cannot exclude the possibility that H89 interferes with the acquisition of other host lipids.

A number of small molecules have been shown to inhibit the intracellular *Chlamydia* infection by reducing inclusion size and, in some cases, infectious progeny (39–45). However, failure to produce progeny could be due to defects at various steps in the developmental cycle, including EB entry, EB-to-RB conversion, RB replication, and RB-to-EB conversion. KSK120, an inhibitor of C. trachomatis glucose metabolism, has been shown to primarily cause a defect in chlamydial replication (46, 47). Studies on other inhibitors that decrease infectious progeny have not distinguished between effects on RB replication and RB-to-EB conversion, where the latter would only decrease the production of EBs but not RBs.

Our studies identified RB-to-EB conversion as the main step in the chlamydial developmental cycle that is inhibited by H89. *Chlamydia*-infected cells treated with H89 could support RB replication, albeit with a 2-fold reduction in total chlamydial numbers. However, the difference between this small replication defect and the 25-fold reduction in infectious progeny indicates a postreplication defect in EB production. Through multiple lines of evidence, we demonstrate that H89 disrupts RB-to-EB conversion. Specifically, we measured decreased expression of EB-specific gene products by Western blotting and microscopy analysis, detected fewer EBs relative to RBs by EM, and observed a reduced number of infectious EBs in progeny assays.

H89 did not interfere with RB-to-EB conversion by inducing chlamydial persistence. Agents such as gamma interferon and penicillin lead to persistence by causing a general block in RB-to-EB conversion with decreased late gene expression and EB production (36, 48). However, a cardinal feature of persistence is a concomitant replication defect that results in very large, nondividing RBs called aberrant bodies (49). In our EM studies of H89-treated cells, there were no aberrant bodies; instead, we only detected normal-appearing RBs and dividing RBs, which is consistent with ongoing RB replication.

H89 may exert its effects on *Chlamydia* infection through its known role as an inhibitor of specific host protein kinases. The best-characterized target of H89 is PKA, which regulates transcription, protein expression, and cell growth and differentiation (50, 51). In Coxiella burnetii, PKA promotes formation of the parasitophorous vacuole and production of infectious progeny (52, 53). However, we found that PKA was not necessary for the C. trachomatis infection and that H89 still altered inclusion size and EB production in PKA knockout cells. PKC is another target of H89 (24, 25). This kinase and its phosphorylated substrates are recruited to the chlamydial inclusion (38). However, H89 did not alter the localization of phosphorylated PKC substrates to the inclusion, suggesting that H89 acts through other kinases, including MSK1, S6K1, PKB, and ROCK-II (25, 27–29). Notably, H89 was shown to reduce intracellular *Salmonella* growth by inhibiting PKB/AKT1 activity (54), but it is not known if these host kinases have roles in inclusion growth and chlamydial development. In addition, H89 might inhibit chlamydial kinases, such as Pkn1, PknD, and Pkn5 (34, 55). Additional studies that are beyond the scope of the manuscript will explore the specific H89 target(s) whose disruption produces the strong phenotypes that we observed.

In conclusion, H89 treatment of *Chlamydia*-infected cells has inhibitory effects on both the chlamydial inclusion and the developmental cycle. Our detailed analysis showed that H89 reduces the production of infectious progeny by causing both a small decrease in RB replication and a much larger defect in RB-to-EB conversion. Conversion from an RB to an EB is a critical step for spreading the intracellular *Chlamydia* infection to a new host cell. However, to the best of our knowledge, no other pharmacological inhibitor has been shown to interrupt RB-to-EB conversion without causing chlamydial persistence. This distinct property makes H89 a useful tool that may lead to new antichlamydial therapeutic strategies.

## MATERIALS AND METHODS

### Pharmacological compounds.

H89 (hydrochloride; CAS 130964-39-5; Cayman Chemical) was reconstituted in dimethyl sulfoxide (DMSO). D/D solubilizer (635054; TakaRa) was used at a final concentration of 1 μM. Brefeldin A (AAJ62340MA; Fisher Scientific) was used at a final concentration of 1 μg/ml.

### Antibodies used in this study.

Primary antibodies used were monoclonal mouse anti-MOMP (generous gift from Ellena Peterson, University of California, Irvine) (56), polyclonal rabbit anti-OmcB (generous gift from Guangming Zhong, UT Health San Antonio) (57), polyclonal rabbit anti-Hc1 and polyclonal rabbit anti-Hc2 (generous gifts from Ted Hackstadt, Rocky Mountain Laboratories, NIAID, NIH, Hamilton, Montana) (58), anti-GM130 (610822; BD Transduction Laboratories), anti-phosphorylated PKC substrates (2261S; Cell Signaling), anti-GAPDH (sc-47724; Santa Cruz), and anti-α-tubulin (T5168; Sigma). Fluorescent secondary antibodies for immunofluorescence microscopy used were donkey anti-rabbit IgG Alexa Fluor 488 (A21206; Invitrogen) and donkey anti-mouse IgG Alexa Fluor 555 (A31570; Invitrogen). Fluorescent secondary antibodies for Western blots were goat anti-rabbit IgG LI-COR IRDye 680 (926-680-71; Fisher Scientific) and goat anti-mouse IgG LI-COR IRDye 800 (926-32210; Fisher Scientific).

### Cell culture and *Chlamydia* infection.

HeLa, RPE-1, and MEF cell lines were purchased from ATCC and cultured at 37°C and 5.0% CO_2_ in Dulbecco’s modified Eagle medium (DMEM) (11995-065; Gibco) supplemented with 10% fetal bovine serum (FBS) (S11550; Atlanta Biologicals). mpkCCD WT and PKA knockout cell lines were a generous gift from Mark Knepper (National Institutes of Health, Bethesda, MD) and were grown in DMEM supplemented with 10% FBS. C1 HeLa cells stably expressing GFP-tagged FM4-hGH were kindly provided by Andrew Peden (University of Sheffield, England). All experiments were conducted in HeLa cells unless stated otherwise.

Monolayers of HeLa cells were infected with C. trachomatis serovar L2, strain L2/434/Bu (ATCC VR-902B), at a multiplicity of infection (MOI) of 3 in SPG (200 mM sucrose, 20 mM sodium phosphate, and 5 mM glutamate, pH 7.2) and centrifuged at 700 × *g* for 1 h at room temperature. After centrifugation, the inoculum was removed and replaced with 500 μl of DMEM supplemented with 10% FBS. C. trachomatis infections in other cell lines were carried out under the same conditions.

### *Chlamydia* infections with inhibitor.

H89 was added to infected monolayers immediately after the 1-h centrifugation step. This inhibitor was present in the medium for the duration of the infection. In replenishment studies, we replaced the medium with fresh medium and fresh inhibitor at 24 hpi.

### Immunofluorescence microscopy.

Cells, grown and infected on glass coverslips, were fixed in either cold 4% paraformaldehyde or 100% ice-cold methanol for 10 min. Cells were permeabilized and incubated in blocking buffer (2% FBS, 0.1% Triton) for 30 min at room temperature. C. trachomatis was visualized with a monoclonal mouse antibody against the major outer membrane protein (MOMP). Coverslips were mounted with ProLong Glass antifade containing NucBlue to stain DNA (P36985; Invitrogen). Immunofluorescence microscopy images were acquired on a Zeiss Axiovert 200M microscope. Inclusion areas were quantified by manual tracing using ImageJ software.

### Viability assay.

Toxic effects of the inhibitor on uninfected host cells were assessed with the MTT assay per the manufacturer’s protocol (ab211091; Abcam).

### Infection efficiency.

HeLa cells infected with *C. trachomatis* at an MOI of 3 were treated with 12.5 μM H89 starting at 1 h postinfection (hpi). At 32 hpi, samples were fixed in ice-cold methanol, permeabilized, blocked, and then stained for MOMP. The number of cells containing an inclusion was quantified from 10 different fields using a 63× objective. The data were expressed as a percentage of cells with an inclusion, which is reflective of infection efficiency.

### qPCR.

The number of chlamydial genomes per infected HeLa cell was measured by qPCR. A plasmid containing the C. trachomatis *euo* gene was used to generate a standard curve from which the *Chlamydia* copy number was calculated. PCRs with primers to the host cell gene GAPDH were performed to generate a PCR product that was used to calculate the total number of host cells. The total number of *Chlamydia genomes* per cell was calculated by normalizing *Chlamydia* copy number (EUO) to the respective GAPDH values. Primer sequences for EUO were 5′-TTATTCCGTGGGACAAGTGG-3′ (forward primer) and 5′-TGCAAGACTTTTCCCTTTGC-3′ (reverse primer). Primer sequences for GAPDH were 5′-GGCGCTCACTGTTCTCTCCC-3′ (forward primer) and 5′-CGCAAGGCTCGTAGACGCG-3′ (reverse primer). Each qPCR utilized SsoAdvanced universal SYBR green supermix (1725271; Bio-Rad) and was run on a Bio-Rad thermocycler.

### Progeny assay.

C. trachomatis-infected cells were treated with H89 starting at 1 hpi. At the indicated time point after infection, cells were washed with 1× PBS, which was then replaced with 500 μl cold SPG. Cells were lysed by freezing at −80°C for 30 min, followed by thawing at 37°C for 15 min and vigorous vortexing. Cell lysates were serially diluted in SPG and used to reinfect fresh monolayers of HeLa cells in the absence of inhibitor. At 27 hpi, cells were fixed in ice-cold methanol, and numbers of inclusion forming units were determined via immunofluorescence microscopy using an antibody to MOMP. The number of inclusions was determined from 10 fields of view observed with a 20× objective. The number of progeny per cell was determined by dividing the total number of infectious progeny (IFU/ml) by the number of host cells present at the start of the infection. The number of host cells was quantified by manual counting of trypsinized cells on a hemocytometer.

### Western blotting.

Cell lysates were prepared by lysing cells directly in 2% SDS (59), followed by boiling of the samples at 95°C for 5 min. Equal volumes of lysate were loaded, separated by SDS-PAGE, and transferred onto nitrocellulose membranes. Membranes were blocked with 5% bovine serum albumin (BSA) in 1× Tris-buffered saline containing 0.1% Tween 20 (TBS-T). The membranes were imaged on an Odyssey CLx Li-COR machine.

### EM.

C. trachomatis-infected HeLa cells were fixed with 2% EM-grade paraformaldehyde (100503-917; VWR) and 2.5% EM-grade glutaraldehyde (NC9861069; Fisher Scientific) for 30 min at room temperature and then overnight at 4°C. Samples were processed and imaged by the Electron Microscopy Core Imaging Facility at the University of Maryland School of Dentistry.

### Protein transport assay.

C1 HeLa cells stably expressing GFP-tagged FM4-hGH were infected with C. trachomatis at an MOI of 3 and grown in the presence or absence of 12.5 μM H89 starting at 1 hpi. Infected cells were treated for up to 100 min with D/D solubilizer at a final concentration of 1 μM, fixed, and analyzed by fluorescence microscopy. As a positive control, infected cells were incubated with 100 μM H89, a concentration known to inhibit protein transport to the cell surface (30), for 2 h prior to solubilizer addition.

### C_6_-NBD ceramide lipid transport assay.

This lipid transport assay was performed as described previously (8). In brief, HeLa cells, seeded on coverslips and infected with C. trachomatis at an MOI of 3, were grown in the presence or absence of 12.5 μM H89. To compare the effect of H89 on lipid transport for inclusions of similar size, control samples were assayed at 24 hpi and H89 samples at 36 hpi. At the indicated time points postinfection, cells were shifted to 4°C for 30 min and washed twice with ice-cold Eagle minimal essential medium (EMEM; 50188268FP; Fisher Scientific). They were then incubated with EMEM supplemented with 0.035% defatted BSA (9048-46-8; Fisher Scientific), 5 μM C_6_-NBD ceramide (NC0339630; Fisher Scientific), and H89. After a 60-min incubation at 4°C, which facilitated C_6_-NBD ceramide labeling, unincorporated C_6_-NBD ceramide was removed by washing twice with EMEM. Cells were then incubated with EMEM containing 0.75% defatted BSA and H89 for the times indicated at 37°C, followed by staining with 4′,6-diamidino-2-phenylindole (DAPI) and mounting for immediate observation by fluorescence microscopy. As a positive control, cells were treated with brefeldin A, which inhibits ceramide transport to the inclusion (19), from 21 to 24 hpi, followed by sample harvesting and analysis at 24 hpi.

### Statistical analysis.

For each experiment, at least 3 independent biological replicates were performed, and results are presented as means ± standard deviations (SD). Data were analyzed by unpaired, two-tailed *t* tests on GraphPad Prism software, version 8.
