# Supplementary material for: The Small Molecule H89 Inhibits Chlamydia Inclusion Growth and Production of Infectious Progeny
Source: Infect Immun. 2021 Jun 16;89(7):e00729-20. doi: 10.1128/IAI.00729-20 (PMC8373235; doi:10.1128/IAI.00729-20)
Supplement: Supplemental file 1 — Fig. S1 to S6. Download IAI.00729-20-s0001.pdf, PDF file, 4.44 MB [file iai.00729-20-s0001.pdf]

Figure S1

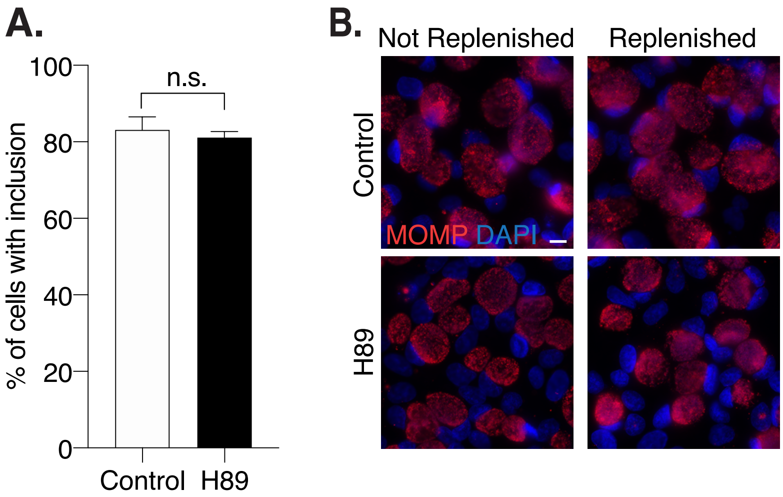

Figure S1. 12.5 µM of H89 does not affect the ability to form inclusions in HeLa cells and is stable during the time course of the intracellular chlamydial infection.
(A) HeLa cells were treated with 12.5 µM of H89 from 1 to 32 hpi. The percentage of HeLa cells with an inclusion was used to determine infection efficiency. n.s.: not statistically significant.
(B) Immunofluorescence images of infected HeLa cells treated with H89 starting at 1 hpi. H89 was either left with the cells for the entire infection or replenished at 24 hpi. Samples were fixed at 48 hpi. Chlamydiae were stained with MOMP (red), while chlamydial and host DNA was detected with DAPI (blue). Scale bar is 10 µm.

Figure S2

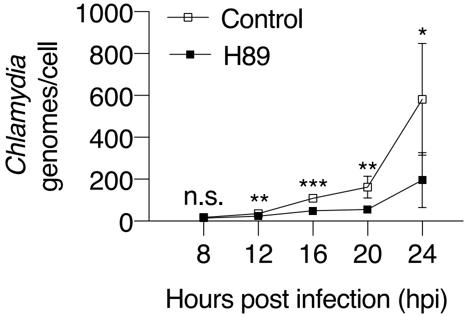

Figure S2. H89 decreases RB replication by 3-fold at 24 hpi during the onset of RB-to-EB conversion.
The number of chlamydial genomes in infected, H89-treated HeLa cells was determined via qPCR at the indicated time points, and normalized to the number of host cells. The data are presented as mean ± SD (n=3); $^{**}P \leq 0.01$, and $^{***}P < 0.001$; n.s.: not statistically significant.

Figure S3

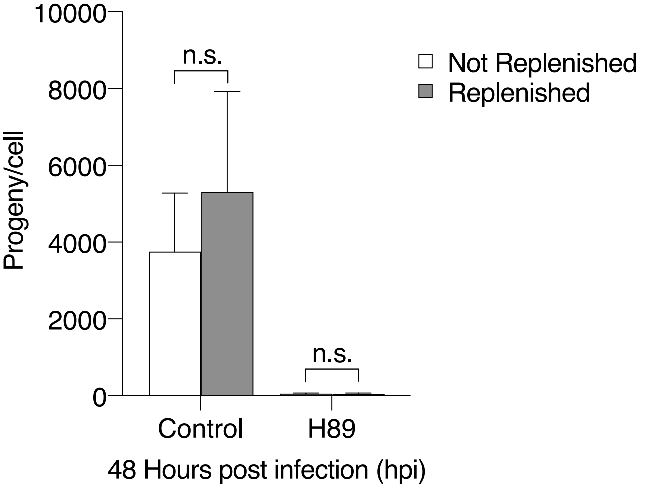

Figure S3. Replenishing H89 does not cause additional inhibitory effects on infectious progeny.
The number of infectious EBs was determined by progeny assay for infected control and H89-treated HeLa cells. H89 was either left with the cells for the entire infection or replenished at 24 hpi, followed by sample collection at 48 hpi. The number of infectious progeny (EBs) per host cell is presented as mean ± SD (n=3); n.s.: not statistically significant.

Figure S4

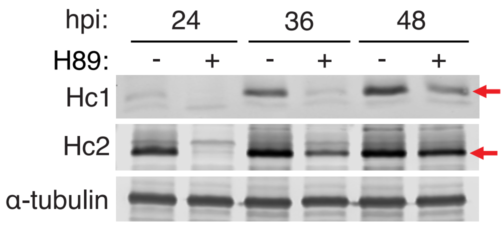

Figure S4. H89 delays expression of two additional chlamydial late genes.
Lysates of *C.trachomatis* L2-infected HeLa cells treated with H89 starting at 1 hpi were analyzed by western blotting at the indicated time points. The levels of histone-like proteins Hc1 and Hc2 (both indicated with a red arrow), as well as the general chlamydial surface protein MOMP are shown. α-tubulin served as a loading control.

Figure S5

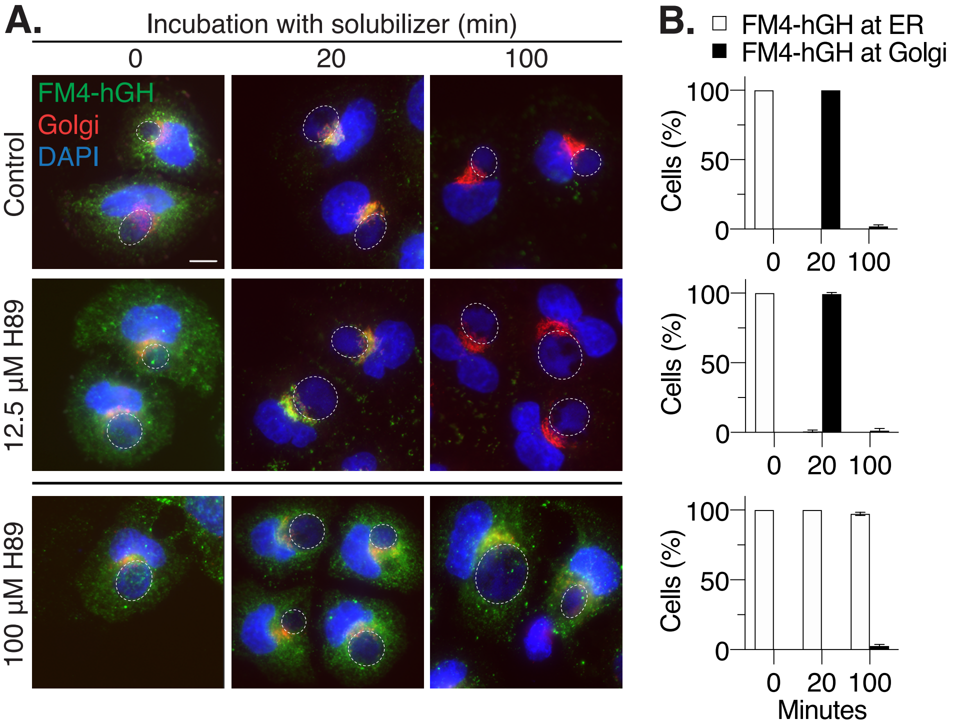

Figure S5. 12.5 µM H89 does not alter ER-to-Golgi or post-Golgi trafficking in *Chlamydia*-infected cells, regardless of inclusion size.

(A) C1 HeLa cells, which express the GFP-tagged transport reporter protein FM4-hGH (green), were infected in the presence of H89 and incubated with solubilizer for 0, 20 or 100 minutes at the indicated time points post infection. To account for differences in inclusion size, we compared the effect of the H89 inhibitor on protein transport for 12.5 µM H89-treated samples at 36 hpi and control samples at 24hpi. The bottom panel shows a control sample in which infected C1 HeLa cells were treated with 100 µM H89 from 22 to 24 hpi, prior to the addition of solubilizer at 24 hpi. The Golgi was visualized with antibodies to GM130 (red), and chlamydial and host DNA were stained with DAPI (blue). Chlamydial inclusions are outlined with white, dashed lines. Scale bar: 10 µm.

(B) The number of cells with GFP-tagged FM4-hGH at either the ER or the Golgi was normalized to the control and expressed as a percentage for the indicated time points post solubilizer addition (minutes). The data are presented as mean ± SD (n=3).

Figure S6

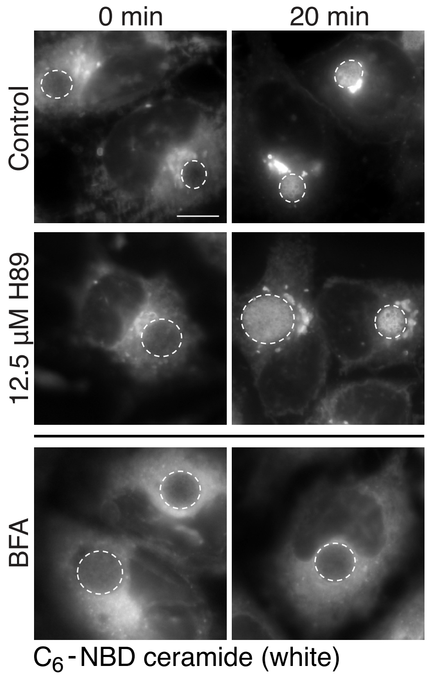

Supplemental Figure 6. 12.5 µM of H89 does not inhibit lipid transport to the inclusion. Fluorescence images of the NBD-ceramide transport assay in *C.trachomatis* L2-infected HeLa cells. Two time points of this assay are shown. To account for differences in inclusion size, we compared NBD-ceramide transport for 12.5 µM H89-treated samples at 36 hpi and control samples at 24hpi. The bottom panel shows a control sample in which infected cells were treated with 5 µM BFA from 21 to 24 hpi prior to ceramide addition. DAPI (not shown) was used to identify the chlamydial inclusions, which are outlined with white, dashed lines. Scale bar: 10 µm.
